# Recurrence of idiopathic acute pancreatitis after cholecystectomy: systematic review and meta‐analysis

**DOI:** 10.1002/bjs.11429

**Published:** 2019-12-25

**Authors:** D. S. Umans, N. D. Hallensleben, R. C. Verdonk, S. A. W. Bouwense, P. Fockens, H. C. van Santvoort, R. P. Voermans, M. G. Besselink, M. J. Bruno, J. E. van Hooft, on behalf of the Dutch Pancreatitis Study Group

**Affiliations:** ^1^ Department of Gastroenterology and Hepatology Amsterdam Gastroenterology and Metabolism, Amsterdam UMC, University of Amsterdam Amsterdam the Netherlands; ^2^ Department of Surgery Amsterdam Gastroenterology and Metabolism, Amsterdam UMC, University of Amsterdam Amsterdam the Netherlands; ^3^ Department of Gastroenterology Erasmus MC University Medical Centre Rotterdam the Netherlands; ^4^ Department of Research and Development St Antonius Hospital Nieuwegein the Netherlands; ^5^ Department of Gastroenterology St Antonius Hospital Nieuwegein the Netherlands; ^6^ Department of Surgery St Antonius Hospital Nieuwegein the Netherlands; ^7^ Department of Surgery MUMC+ Maastricht the Netherlands; ^8^ Department of Surgery UMC Utrecht Utrecht the Netherlands

## Abstract

**Background:**

Occult biliary disease has been suggested as a frequent underlying cause of idiopathic acute pancreatitis (IAP). Cholecystectomy has been proposed as a strategy to prevent recurrent IAP. The aim of this systematic review was to determine the efficacy of cholecystectomy in reducing the risk of recurrent IAP.

**Methods:**

PubMed, Embase and Cochrane Library databases were searched systematically for studies including patients with IAP treated by cholecystectomy, with data on recurrence of pancreatitis. Studies published before 1980 or including chronic pancreatitis and case reports were excluded. The primary outcome was recurrence rate. Quality was assessed using the Newcastle–Ottawa Scale. Meta‐analyses were undertaken to calculate risk ratios using a random‐effects model with the inverse‐variance method.

**Results:**

Overall, ten studies were included, of which nine were used in pooled analyses. The study population consisted of 524 patients with 126 cholecystectomies. Of these 524 patients, 154 (29·4 (95 per cent c.i. 25·5 to 33·3) per cent) had recurrent disease. The recurrence rate was significantly lower after cholecystectomy than after conservative management (14 of 126 (11·1 per cent) *versus* 140 of 398 (35·2 per cent); risk ratio 0·44, 95 per cent c.i. 0·27 to 0·71). Even in patients in whom IAP was diagnosed after more extensive diagnostic testing, including endoscopic ultrasonography or magnetic resonance cholangiopancreatography, the recurrence rate appeared to be lower after cholecystectomy (4 of 36 (11 per cent) *versus* 42 of 108 (38·9 per cent); risk ratio 0·41, 0·16 to 1·07).

**Conclusion:**

Cholecystectomy after an episode of IAP reduces the risk of recurrent pancreatitis. This implies that current diagnostics are insufficient to exclude a biliary cause.

## Graphical abstract

Idiopathic acute pancreatitis recurs more often than acute pancreatitis of known cause. In at least some of these patients, standard diagnostics (laboratory tests, transabdominal ultrasonography) may fail to detect biliary disease. The literature on cholecystectomy as a preventive measure for recurrence of pancreatitis in presumed idiopathic acute pancreatitis was reviewed systematically.

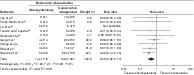

Cholecystectomy can reduce pancreatitis

## Abstract (es)

**Antecedentes:**

Se ha sugerido que la enfermedad biliar oculta es una causa subyacente frecuente de pancreatitis aguda idiopática (*idiopathic acute pancreatitis*, IAP). La colecistectomía se ha propuesto como una estrategia para prevenir la IAP recidivante. El objetivo de esta revisión sistemática era determinar la eficacia de la colecistectomía para reducir el riesgo de la IAP recidivante.

**Métodos:**

Se realizó una búsqueda sistemática en PubMed, Embase y Cochrane de estudios que incluían pacientes con IAP tratados con colecistectomía, y con datos sobre la recidiva de la pancreatitis. Se excluyeron los estudios anteriores a 1980, los que incluían pancreatitis crónica y los casos clínicos. El resultado principal fue la tasa de recidiva. La calidad se evaluó utilizando la escala de Newcastle‐Ottawa. Se realizaron metaanálisis para calcular la tasa de riesgo utilizando un modelo de efectos aleatorios con el método de varianza inversa.

**Resultados:**

En total, se incluyeron 10 estudios, de los cuales 9 se utilizaron para realizar análisis agrupados. La población de estudio incluyó 524 pacientes en los que se habían efectuado 126 colecistectomías. De estos 524 pacientes, 154 (29% (i.c. del 95% 25,5‐33,3)) presentaron recidiva de la enfermedad. La tasa de recidiva fue significativamente menor después de la colecistectomía que después del tratamiento conservador (14/126 (11%) *versus* 140/398 (35)); tasa de riesgo 0,44 (i.c. del 95% 0,27‐0,71)). Incluso en pacientes en los que se diagnosticó IAP tras haber efectuado pruebas diagnósticas más extensas, incluyendo ultrasonografía endoscópica o colangiopancreatografía por resonancia magnética, la tasa de recidiva después de la colecistectomía era menor (4/36 (11%) *versus* 42/108 (39%); tasa de riesgo 0,41 (i.c. del 95% 0,16‐1,07)).

**Conclusión:**

La práctica de una colecistectomía después de un episodio de IAP disminuye el riesgo de pancreatitis recidivante. Esto implica que los diagnósticos actuales son insuficientes para excluir una causa biliar (PROSPERO CRD42017055275).

## Introduction

Acute pancreatitis is an increasing healthcare problem^1^ with a wide range of causes. A biliary cause is found in approximately half of patients, followed by alcohol consumption in approximately 20 per cent and less common causes such as medication, hypertriglyceridaemia and autoimmune diseases. In as many as one‐third of patients, the aetiology of acute pancreatitis remains unknown (initially), and the disease is referred to as idiopathic acute pancreatitis (IAP)^2^, ^3^.

Numerous studies have suggested that microlithiasis and sludge might cause a large subset of IAP^4^, ^5^. Small stones (less than 4 mm), usually referred to as microlithiasis^6^, and sludge are often difficult to detect by transabdominal ultrasound imaging, especially if located in the common bile duct (CBD). Therefore, in daily practice, many patients who are initially thought to have IAP may, in fact, have biliary pancreatitis. Gallstones, microlithiasis and sludge are all considered as potential biliary causes of pancreatitis. To reduce the risk of recurrent acute pancreatitis, same‐admission cholecystectomy is advised for mild biliary pancreatitis^7^.

Some studies^8^, ^9^ have advised cholecystectomy after acute pancreatitis if no other aetiology can be found implying the diagnosis of IAP during evaluation. However, the work‐up for a potential biliary cause in these studies was incomplete. Endoscopic ultrasound imaging (EUS), which has been shown to detect a biliary aetiology in one‐third of patients with IAP, and, to a lesser extent, magnetic resonance cholangiopancreatography (MRCP), were often not done^10^.

The primary aim of this systematic review was to determine the efficacy of cholecystectomy in reducing the recurrence rate of pancreatitis in patients with IAP. Patients with presumed IAP and those in whom IAP remained the most likely diagnosis after extensive evaluation were analysed separately.

## Methods

This review was written in accordance with PRISMA^11^ and MOOSE guidelines^12^, and was registered in the PROSPERO database (CRD42017055275).

### Definitions

Data were analysed based on the definitions of IAP as outlined in the original articles, and according to current guidelines^13^, which define IAP as acute pancreatitis in which no aetiology can be determined by standard diagnostic evaluation, consisting of a detailed history, laboratory serum tests (liver enzymes, calcium and triglycerides) and imaging (transabdominal ultrasonography on admission and repeated after discharge).

**Figure 1:**
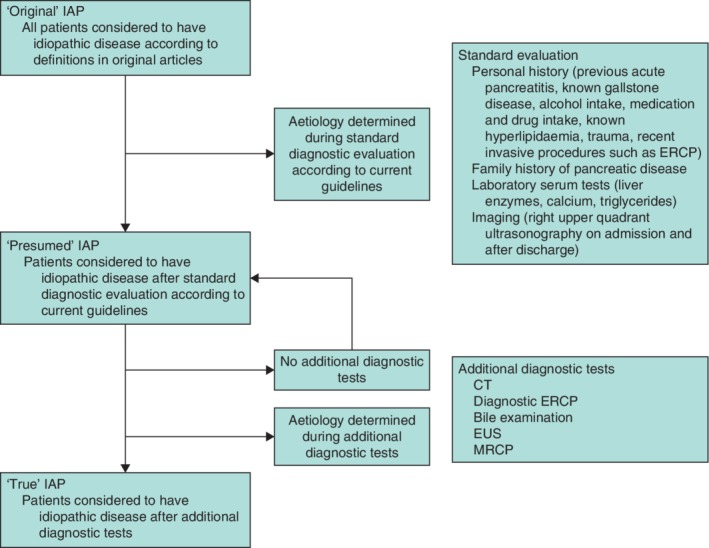
Diagnostic process and definitions IAP, idiopathic acute pancreatitis; ERCP, endoscopic retrograde cholangiopancreatography; EUS endoscopic ultrasonography; MRCP, magnetic resonance cholangiopancreatography.

Three types of IAP were defined for the purposes of this study. First, ‘original’ IAP was defined in accordance with definitions used in the original articles. Second, ‘presumed’ IAP was defined by diagnosis of IAP after the standard evaluation. Third, ‘true’ IAP was defined as an acute pancreatitis episode that remained unexplained after both standard diagnostic work‐up and additional diagnostic tests such as EUS and MRCP (*Fig*. 1).

### Outcome measures

The primary outcome was recurrence rate of acute pancreatitis, calculated as the proportion of patients experiencing one or multiple episodes of recurrent acute pancreatitis after an index episode of ‘original’, ‘presumed’ or ‘true’ IAP.

Secondary outcomes were complications of cholecystectomy, severity of recurrences as defined by the revised Atlanta classification^14^, and occurrence of biliary events before cholecystectomy.

### Search strategy

Guided by an experienced librarian, the PubMed, Embase and Cochrane Library databases were searched systematically for relevant articles published between inception and 1 September 2018 (*Appendix* *S1*, supporting information). Search terms included ‘pancreatitis’, ‘idiopathic’ and ‘cholecystectomy’. Studies of adult humans in English were considered. Duplicates were removed and the search results were recorded using the Covidence systematic review software (Veritas Health Innovation, Melbourne, Victoria, Australia).

### Study selection

Two reviewers screened potentially relevant articles independently by examining the titles and abstracts. Studies were included if they fulfilled the following criteria: the study cohort comprised patients with IAP; the intervention was cholecystectomy and the comparator conservative treatment; and the outcome was rates of recurrent acute pancreatitis. Exclusion criteria were: letters, comments, case reports, reviews, conference abstracts, book chapters, studies not written in English, and studies published before 1980, owing to discrepancies in diagnostic evaluation before 1980 compared with current state‐of‐the‐art work‐up.

The two reviewers read the full text of potentially eligible studies individually. The reference lists of included articles were screened for relevant publications not identified by the initial search. Disagreements regarding eligibility were resolved after joint re‐evaluation by the two reviewers.

### Data extraction

After selecting studies that met the inclusion criteria, all relevant data from these studies were extracted by two reviewers using a standard form. Relevant data included: study characteristics (authors, years of inclusion, publication year, country, study design, number of patients, duration of follow‐up), patient characteristics (sex, age, recurrent or first episode of pancreatitis, number of previous attacks, severity of pancreatitis, previous cholecystectomy), diagnostic evaluation (history, laboratory tests, imaging), interventions (cholecystectomy) and outcome measures. No attempt was made to communicate with the corresponding authors concerning missing data. Missing information was registered as ‘not reported’ and studies with missing data were excluded from subsequent pooled analyses.

### Quality assessment

Two reviewers appraised the quality of the included studies independently using the Newcastle–Ottawa Scale for cohort studies^15^. In tailoring the scale for the purpose of this review, presence of sludge as an exclusion criterion for the intervention and comparator groups was considered to be the most important factor indicating comparability between these groups. Other relevant factors were CBD width, raised serum alanine aminotransferase (ALT) levels, and cholecystectomy before index admission. Follow‐up of at least 2 years was considered to be adequate for recurrence to have occurred. Loss to follow‐up exceeding 10 per cent was considered likely to introduce bias. Disagreement was resolved after discussion between the two reviewers.

### Statistical analysis

Study characteristics, patient characteristics, use of diagnostic tests, treatment with cholecystectomy and secondary outcome measures were reported descriptively.

Pooled recurrence rates from the included studies were reported as proportions and percentages, with two‐sided 95 per cent confidence intervals. Recurrence rates were pooled in meta‐analysis using a random‐effects model with the inverse‐variance method to calculate risk ratios with 95 per cent confidence intervals. Subgroup analyses of patients with ‘presumed’ IAP and ‘true’ IAP were undertaken. Statistical between‐study heterogeneity was assessed using the *I* ^2^ statistic. *I* ^2^ values of less than 25 per cent, 25–49 per cent, 50–75 per cent and more than 75 per cent were considered to indicate low, moderate, high and very high levels of heterogeneity respectively^16^. To evaluate publication bias, a funnel plot was created using Egger's linear regression method^17^, ^18^.

## Results

### Study selection

**Figure 2:**
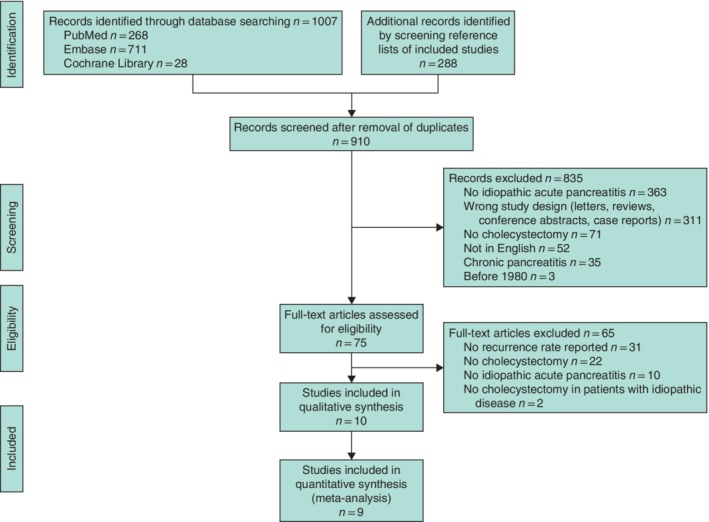
PRISMA flow chart showing selection of articles for review

From PubMed (268 records), Embase (711) and Cochrane Library (28) searches, with additional records identified through screening of reference lists (288), ten articles were selected for inclusion in the qualitative analysis. One case–control study^19^ included a highly selected group of 23 patients who eventually underwent cholecystectomy. Considering potential selection bias, this study was excluded from the quantitative analyses, leaving nine studies in the meta‐analyses (*Fig*. 2).

### Study characteristics

Among the ten included studies, there was one RCT^8^, one cross‐sectional study^20^, six prospective cohort studies^4^, ^5^, ^21^, ^22^, ^23^, ^24^ and two^9^, ^19^ retrospective cohort studies (*Table* 1). The only RCT^8^ compared cholecystectomy with conservative treatment in 85 patients with IAP, with an allocation ratio of 1 : 1. The person enrolling patients in the trial was blinded to the treatment allocation, before block randomization. Patients, physicians and researchers were not blinded. EUS was not used in this RCT, which enrolled patients between January 2009 and January 2013.

**Table 1:** Characteristics of included studies

Reference	Inclusion period	Country	Study design	No. of patients	Follow‐up (months)*
Lee *et al*.^4^	1980–1988	New Zealand, USA	Prospective cohort study	86	48 (6–84)
Pérez‐Martín *et al*.^20^	1994–1996	Spain	Observational transverse cohort study	18	n.r.
Liu *et al*.^22^	1996–1997	China	Prospective cohort study	89	22†
Tandon and Topazian^24^	n.r.	USA	*Post hoc* analysis of prospective database	41	16 (4–44)
Saraswat *et al*.^21^	n.r.	India	Prospective cohort study	24	30 (4–48)
Garg *et al*.^5^	1995–2003	India	Prospective cohort study	75	17·6 (1–156)
Ortega *et al*.^23^	2005–2009	Spain	Prospective cohort study	49	16(9)‡
Trna *et al*.^19^	1990–2005	USA	Retrospective case–control study	239	99 (8–220)†
Räty *et al*.^8^	2009–2013	Finland	RCT	85	36 (5–58)†
Stevens *et al*.^9^	2005–2015	Australia	Retrospective cohort study	195	50 (6)§

* Values are mean (range) unless indicated otherwise; values are

† median (range),

‡ mean(s.d.) and

§ mean (minimum). n.r., Not reported.

### Patient characteristics

In total, 901 patients with acute pancreatitis were included. Among these patients, the cause was biliary in 325, alcoholic in 16, known but unspecified in ten^24^, hyperlipidaemia in two and a duodenal duplication cyst in one patient. A total of 547 patients were considered to have ‘original’ IAP. Of these, 23 patients were included in one case–control study^19^ and were excluded from further analyses, leaving 524 patients with ‘original’ IAP in the meta‐analysis.

Six cohorts^5^, ^19^, ^20^, ^21^, ^23^, ^24^ included patients with recurrent IAP, whereas three studies^4^, ^9^, ^22^ did not report this. Only one study^8^ excluded patients with a recurrent episode of ‘presumed’ IAP (*Table* 2).

**Table 2:** Characteristics of included patients with idiopathic acute pancreatitis

Reference	No. of patients with IAP	Male	Age (years)*	Recurrent pancreatitis	No. of previous attacks*	Severe pancreatitis	Previous cholecystectomy
Lee *et al*.^4^	29§	16 (55)	53 (31–79)	n.r.	n.r.	n.r.	0 (0)
Pérez‐Martín *et al*.^20^	18	8 (44)	54	5 (28)	1 (4 patients) and 3 (1 patient)	4 (22)¶	0 (0)
Liu *et al*.^22^	18	9 (50)	68 (24–86)†	n.r.	n.r.	n.r.	0 (0)
Tandon and Topazian^24^	31	12 (39)	48·8 (19–87)	17 (55)	44 in 17 patients	n.r.	3 (10)
Saraswat *et al*.^21^	24	4 (17)	36 (18–56)	24 (100)	4 or more	n.r.	0 (0)
Garg *et al*.^5^	75	60 (80)	31·9 (14–67)	75 (100)	4·82 (2–10)	n.r.	n.r.
Ortega *et al*.^23^	49	24 (49)	58(17)‡	16 (33)	n.r.	5 (10)#	9 (18)
Trna *et al*.^19^	23	10 (43)	n.r.	8 (35)	2 (6 patients) and 3 (2 patients)	n.r.**	0 (0)
Räty *et al*.^8^	85	52 (61)	Intervention group 56† Control group 57†	0 (0)	–	4 (5)††	0 (0)
Stevens *et al*.^9^	195	100 (51·3)	54 (15–93)†	n.r.	n.r.	n.r.	0 (0)
Total	547	295 (53·9)	–	145	–	13	12

Values in parentheses are percentages unless indicated otherwise;

* values are mean (range), except

† median (range) and

‡ mean(s.d.).

§ Two of 31 patients initially considered to have idiopathic acute pancreatitis (IAP) were later found to have a dilated common bile duct on CT and endoscopic retrograde cholangiopancreatography, and subsequently excluded from analysis.

¶ Based on Ranson criteria.

# Based on Atlanta classification.

** Trna *et al*. reported 40 patients with severe pancreatitis in the entire cohort but did not specify severity in IAP subgroup.

†† Based on revised Atlanta classification. n.r., Not reported.

### Critical appraisal

Most of the studies scored 3^20^, ^24^, 4^5^, ^21^, ^22^, ^23^ or 5^4^, ^19^ of a maximum of 9 points on the Newcastle–Ottawa Scale. One study^9^ scored 6 points and the RCT^8^ scored 8 points. Nearly all studies had trouble ensuring comparability between cohorts. Only one study^19^ controlled for the presence of sludge, raised liver enzyme levels and cholecystectomy before index admission (*Fig*. *S1* and *Table* *S1*, supporting information). A funnel plot of the included studies showed a symmetrical plot, making publication bias highly unlikely (*Figs* *S2* and *S3*, supporting information).

### Diagnostic evaluation

The definition of IAP varied widely among the included studies. None of the studies reported use of standard diagnostic work‐up as described in the International Association of Pancreatology/American Pancreatic Association guideline^13^ to determine the most likely aetiology. Most notably, definitions of alcoholic and biliary aetiology varied broadly between studies (*Table* *S2*, supporting information). Two studies^19^, ^21^ excluded patients based on raised levels of liver enzymes. Although all studies considered cholelithiasis on imaging to be an exclusion criterion for IAP, four^19^, ^21^, ^23^, ^24^ did not require ultrasonography in all patients or did not mention which imaging modality was used. One study^9^ included patients with raised ALT levels, and another^8^ included patients with raised levels of liver enzymes, but only if MRCP was negative for CBD stones. Only five studies considered CBD dilatation^4^, ^20^ or presence of biliary sludge on imaging^5^, ^9^, ^20^, ^24^ to be indicative of biliary aetiology. One study^9^ reported explicitly on the presence of biliary sludge on transabdominal ultrasound imaging, but chose to consider this as indicative of IAP. Repeat transabdominal ultrasonography was commonly employed; five studies^4^, ^5^, ^8^, ^20^, ^21^ used it in all included patients, and two^22^, ^24^ in part of the cohort.

Eighteen of the 524 patients (3·4 per cent) with ‘original’ IAP appeared to have a demonstrable aetiology after review of the results of standard work‐up; the disease was classified as ‘presumed’ IAP in the remaining 506 patients. Additional diagnostic testing comprised CT^4^, ^5^, ^8^, ^22^, ^24^, endoscopic retrograde cholangiopancreatography^4^, ^5^, ^21^, ^22^, ^24^, microscopic bile examination^4^, ^5^, ^20^, ^21^, ^23^, ^24^, EUS^5^, ^22^, ^23^, ^24^ and MRCP^8^, ^9^, ^23^, ^24^. Additional diagnostic tests demonstrated biliary disease in 25·8 per cent (111 patients), chronic pancreatitis in 15·2 per cent (47; although only 1 study^23^ reported diagnostic criteria for chronic pancreatitis), pancreatic divisum in 3·9 per cent (12), neoplasms in 1·3 per cent (4) and ascariasis, choledochal cyst and choledochocele in 0·3 per cent (1). In total, a previously unknown potential cause of acute pancreatitis was found using additional tests in 165 patients (32·6 per cent) (*Table* *S3*, supporting information).

### Cholecystectomy

Of 524 patients with ‘original’ IAP, 126 (24·0 per cent) underwent cholecystectomy during follow‐up. To create a subgroup of patients with ‘true’ IAP, several groups of patients were excluded: those in whom an aetiology was established during either standard (18) or additional (165) work‐up, those for whom it was not sufficiently reported whether biliary disease was present (195)^9^ and patients in whom the disease course during follow‐up was unclear (2)^23^. In the subgroup of 144 patients with ‘true’ IAP, 36 cholecystectomies (25·0 per cent) were performed (*Fig*. *S4*, supporting information).

One study^8^ also reported pathology results for the gallbladder. Microlithiasis was observed on pathological examination in 23 of 39 gallbladders.

#### *Complications of cholecystectomy*

One study^9^ reported one bile duct injury in 66 cholecystectomies, and two studies^8^, ^22^ reported no complications in 13 and 39 cholecystectomies respectively. In total, there was one complication in 118 cholecystectomies (0·8 (95 per cent c.i. 0 to 2·5) per cent). Cholecystectomy complication rates were not reported in the remaining studies.

### Recurrence

**Figure 3:**
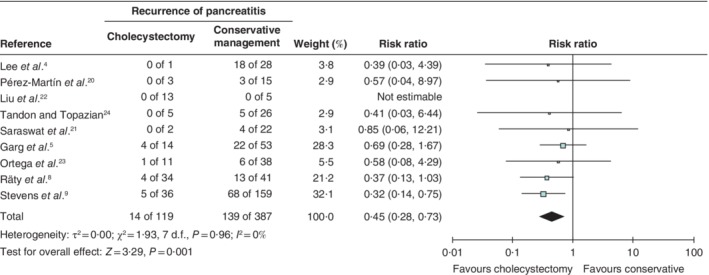
Pooled analysis of recurrence of pancreatitis in patients with ‘presumed’ idiopathic acute pancreatitis treated with cholecystectomy *versus* conservative management Risk ratios are shown with 95 per cent confidence intervals. A random‐effects inverse‐variance model was used for meta‐analysis.

Of the 524 patients with ‘original’ IAP, 154 had at least one recurrence during follow‐up (29·4 (95 per cent c.i. 25·5 to 33·3) per cent). Meta‐analysis of this group showed that the recurrence rate among patients managed conservatively was significantly higher than that in patients who underwent cholecystectomy (140 of 398 (35·2 per cent) *versus* 14 of 126 (11·1 per cent); risk ratio 0·44, 95 per cent c.i. 0·27 to 0·71) (*Fig*. *S5*, supporting information). Similarly, in the subgroup of 506 patients with ‘presumed’ IAP, the recurrence rate was higher among patients who received conservative treatment (139 of 387 (35·9 per cent) *versus* 14 of 119 (11·8 per cent); risk ratio 0·45, 0·28 to 0·73) (*Fig*. 3).

**Figure 4:**
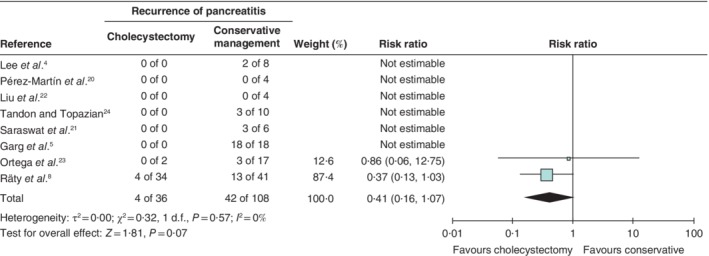
Pooled analysis of recurrence of pancreatitis in patients with ‘true’ idiopathic acute pancreatitis treated with cholecystectomy *versus* conservative management Risk ratios are shown with 95 per cent confidence intervals. A random‐effects inverse‐variance model was used for meta‐analysis.

Among 144 patients with ‘true’ IAP, 46 had at least one recurrence during follow‐up (31·9 (30·8 to 46·8) per cent). In pooled analysis, the recurrence rate was 11 per cent (4 of 36) in the cholecystectomy group and 38·9 per cent (42 of 108 patients) in the conservative treatment (risk ratio 0·41, 0·16 to 1·07) (*Fig*. 4).

There was no statistical between‐study heterogeneity in any of the pooled analyses (*I* ^*2*^ = 0 per cent).

None of the included studies reported severity of recurrences.

### Biliary events before cholecystectomy

The occurrence of biliary events (cholecystitis, biliary colic, obstructive choledocholithiasis, biliary pancreatitis and cholangitis) was not reported systematically. Three studies briefly mentioned biliary events before cholecystectomy. One study^22^ reported no biliary events, and another^20^ reported one patient with a recurrent episode of acute (biliary) pancreatitis, after which cholecystectomy was performed. The third study^4^ reported 13 patients with recurrent episodes of biliary pancreatitis, five of whom were treated by cholecystectomy and three by endoscopic sphincterotomy.

## Discussion

This systematic review and meta‐analysis showed that cholecystectomy might reduce the risk of recurrence of IAP. This effect appeared to be independent of the evaluation before making the diagnosis of IAP.

The efficacy of cholecystectomy in preventing biliary events after biliary pancreatitis is undisputed^7^. The results of this review are therefore in line with the theory that a significant number of patients with ‘presumed’ and ‘true’ IAP actually have biliary pancreatitis. This is exemplified by the high rate of microlithiasis on pathological examination of the gallbladder^8^. Previous research^10^ has suggested that additional diagnostic work‐up with EUS and MRCP may detect a biliary cause in patients with IAP, after negative transabdominal ultrasonography and biochemical tests. In the present study, however, the impact of cholecystectomy in reducing recurrence of acute pancreatitis appeared to be independent of the preoperative evaluation, either including or excluding MRCP and EUS. Possible explanations for this are the suboptimal sensitivity of MRCP for the detection of sludge and lack of a standardized approach to EUS.

Another intriguing finding is the larger number of other pancreatic disorders observed in the included studies, apart from biliary disease. Most notably, chronic pancreatitis was diagnosed in 15·2 per cent and neoplasms in 1·3 per cent. Additionally, pancreas divisum was found in 12 patients (3·9 per cent), although a causative relationship between pancreas divisum and acute pancreatitis is debated^25^.

The present results should be interpreted in light of several shortcomings. First, most of the included studies were small in size, especially the subgroup of the 144 patients with ‘true’ IAP, in whom only 36 cholecystectomies were performed. This subgroup analysis showed no significant difference in recurrence rate after cholecystectomy, possibly owing to insufficient sample size.

Second, there was heterogeneity between studies as some included both patients with a first episode of IAP and those with recurrent IAP, and definitions of IAP differed across studies. Partly owing to evolving insights regarding work‐up of IAP and availability of diagnostic tests, many of the included studies did not undertake complete standard and additional diagnostic testing according to current international guidelines^13^. This may have led to the inclusion of patients in whom a biliary aetiology could have been demonstrated if standard and additional diagnostic tests had been carried out properly. Including those in whom biliary disease went undiagnosed may have led to overestimation of the effect of cholecystectomy in IAP.

Third, only one study^8^ had a randomized design, but this trial was not sham‐controlled and the patients were not blinded. Undergoing surgery may influence the patient's lifestyle, and previous literature^26^ has shown that cessation of alcohol and nicotine use are particularly effective in preventing recurrence.

Fourth, cholecystectomy was almost always undertaken only in patients with proven biliary disease after additional investigation. Only one study^23^ that performed EUS, and one^8^ that performed MRCP if indicated in 28 patients, undertook cholecystectomies in patients with ‘true’ IAP (*Fig*.  4). This confounding by indication creates a clear overestimation of the effect of cholecystectomy. In the most relevant subgroup studied in this review, patients with ‘true’ IAP, this overestimation is reduced to an important extent.

Future studies should address discrepancies in defining IAP as opposed to biliary pancreatitis. Reaching international consensus regarding the criteria for diagnosis of aetiologies is desirable, and would facilitate unambiguity in research as well as in clinical practice. A guideline‐based proposal of such criteria is provided in *Fig*. *S6* (supporting information). Future studies in IAP should also focus on patients with either a first episode of IAP or recurrent pancreatitis, as these two groups appear to have distinct disease courses and should be considered as separate entities^27^.

This review has shown that cholecystectomy could potentially reduce the recurrence rate in patients diagnosed with ‘true’ IAP. However, the results for this subgroup were not statistically significant, probably because of the relatively small sample size. Thus, there appears to be some merit in treating IAP pragmatically by cholecystectomy to prevent recurrence, as suggested in previous studies^8^, ^9^. On the other hand, with further standardization and improvement of diagnostic work‐up, it should be possible to identify most patients with biliary aetiology. The wide variety of aetiologies revealed by additional investigation in the included studies underlines the value of additional diagnostic tests, at least in recurrent idiopathic pancreatitis. More research is needed to determine the importance of routine additional diagnostic work‐up and to establish whether the yield of extra information could outweigh the efficacy of a pragmatic cholecystectomy in preventing recurrence.

The present review supports the hypothesis that many patients with IAP have occult biliary disease by showing an apparent reduction in recurrence after cholecystectomy in patients in whom no additional preoperative biliary diagnostics were undertaken. This underlines the need for a more thorough evaluation before the diagnosis of IAP can be made. Additional research is needed in patients with ‘true’ IAP after optimal testing for biliary aetiology to determine the efficacy of cholecystectomy in this specific population.

## Supporting information


**Fig. S1.** Critical appraisal according to Newcastle–Ottawa Scale
**Fig. S2.** Odds ratio analysis of all included studies
**Fig. S3.** Funnel plot
**Fig. S4.** Overview of cholecystectomies performed
**Fig. S5.** Recurrence rate in original IAP patients managed by cholecystectomy *versus* conservatively or by other treatment
**Fig. S6.** Protocol for diagnosis and criteria for diagnosis of aetiological factors

**Appendix S1.**. Supporting Information.

**Table S1.** Assessment of quality according to Newcastle–Ottawa Scale

**Table S2.** Components of standard work‐up according to current guidelines and extent to which they were executed in included studies

**Table S3.** Additional diagnostic work‐up
