# Supplementary material for: Recurrence of idiopathic acute pancreatitis after cholecystectomy: systematic review and meta‐analysis
Source: Br J Surg. 2019 Dec 25;107(3):191–9. doi: 10.1002/bjs.11429 (PMC7003758; doi:10.1002/bjs.11429)
Supplement: Supplementary file 1 — Fig. S1. Critical appraisal according to Newcastle–Ottawa Scale Fig. S2. Odds ratio analysis of all included studies Fig. S3. Funnel plot Fig. S4. Overview of cholecystectomies performed Fig. S5. Recurrence rate in original IAP patients managed by cholecystectomy versus conservatively or by other treatment Fig. S6. Protocol for diagnosis and criteria for diagnosis of aetiological factors [file BJS-107-191-s001.docx]

**BJS11429**

# Systematic review and meta-analysis of recurrence of idiopathic acute pancreatitis after cholecystectomy

D. S. Umans, N. D. L. Hallensleben, R. C. Verdonk, S. A. W. Bouwense, P. Fockens, H. C. van Santvoort, R. P. Voermans, M. G. Besselink, M. J. Bruno and J. E. van Hooft, on behalf of the Dutch Pancreatitis Study Group

**Appendix S1 Search strategies**

**Table S1 Assessment of quality according to Newcastle–Ottawa Scale**

**Table S2 Components of standard work-up according to current guidelines and extent to which they were executed in included studies**

**Table S3 Additional diagnostic work-up**

**<Appendix S1 and Tables S1–S3 are provided as pdf files>**

**Fig. S1 Critical appraisal according to Newcastle–Ottawa Scale**

**
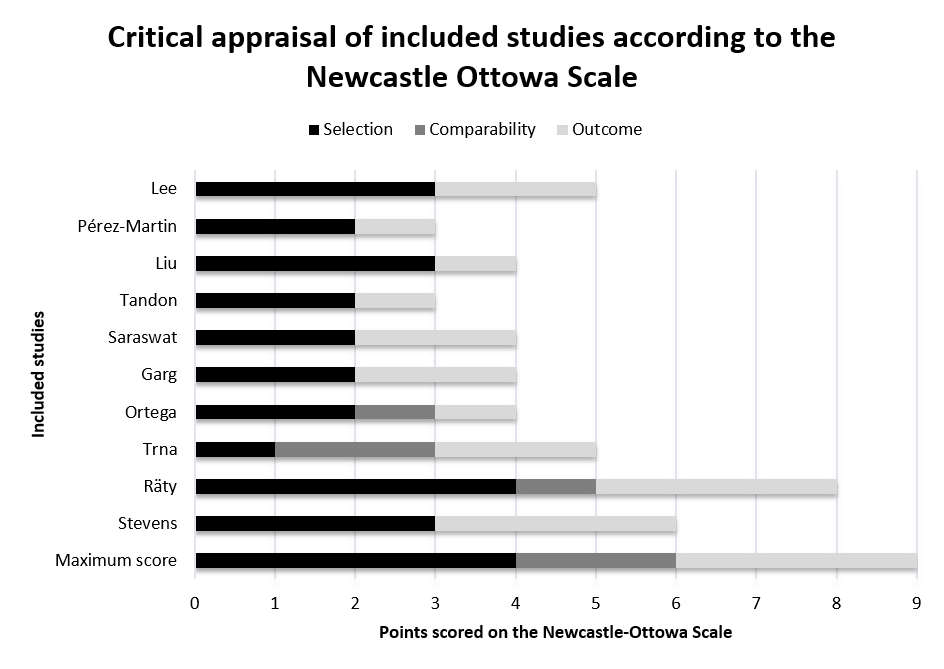
**

**Fig. S2 Odds ratio analysis of all included studies**

**
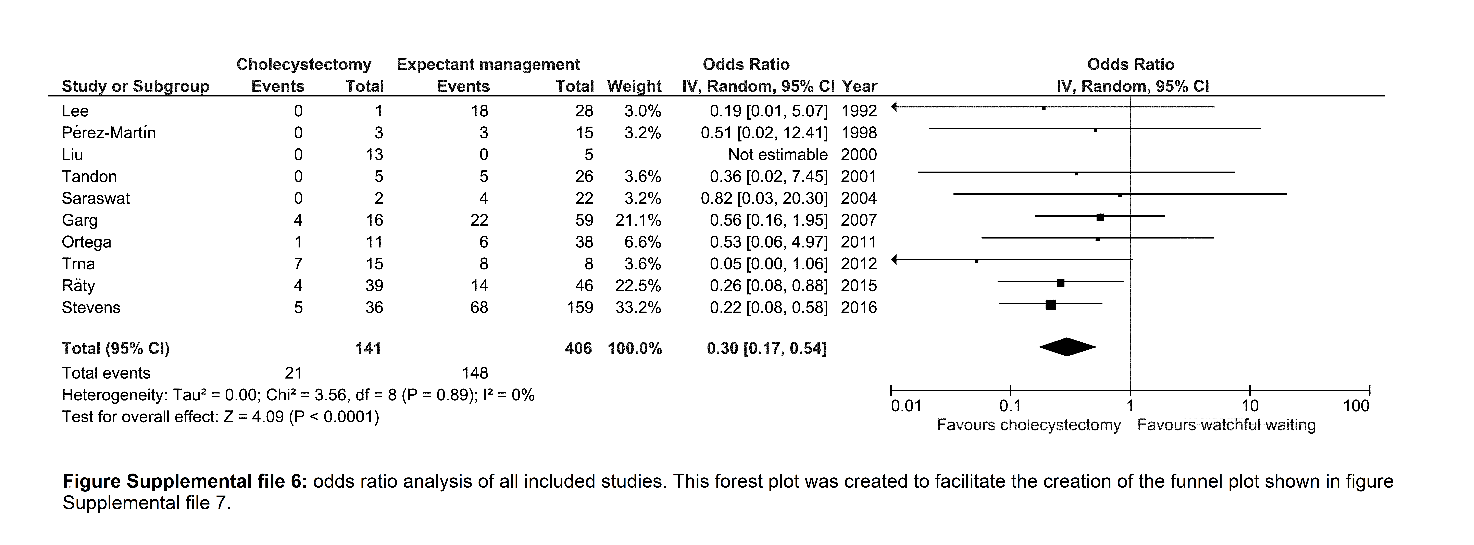
**

**Fig. S3 Funnel plot**

**

**

**Fig. S4 Overview of cholecystectomies performed**

**

**

**Fig. S5 Recurrence rate in original IAP patients managed by cholecystectomy *versus*  conservatively or by other treatment**

**
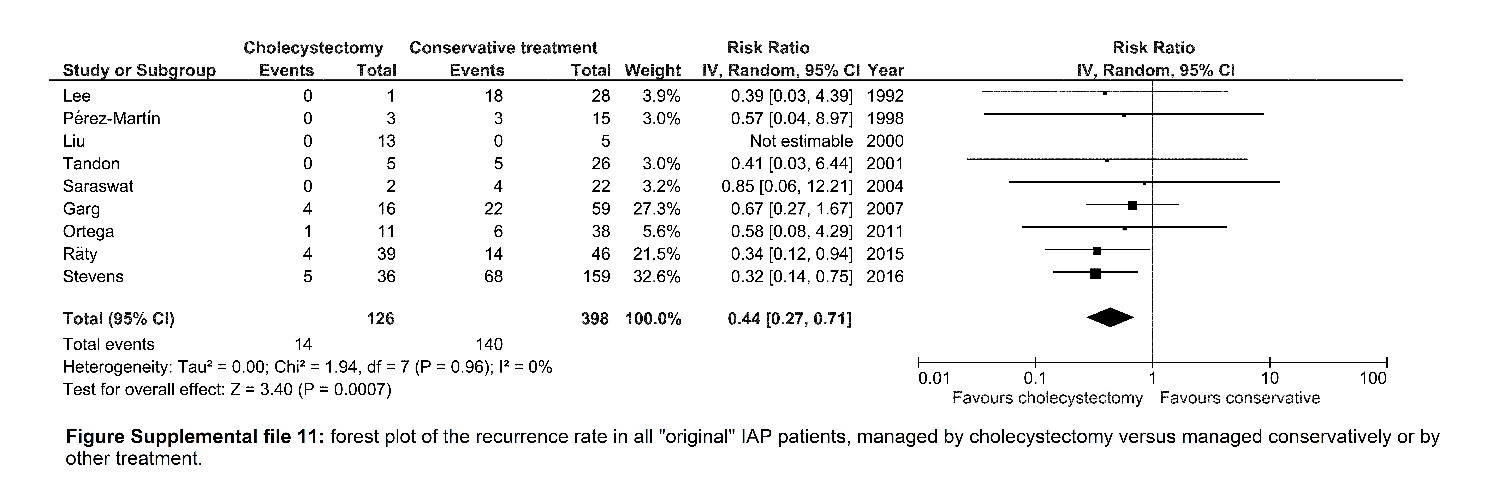
**

**Fig. S6 Protocol for diagnosis and criteria for diagnosis of aetiological factors**

**

**
