# Supplementary material for: Recurrence of idiopathic acute pancreatitis after cholecystectomy: systematic review and meta‐analysis
Source: Br J Surg. 2019 Dec 25;107(3):191–9. doi: 10.1002/bjs.11429 (PMC7003758; doi:10.1002/bjs.11429)
Supplement: Supplementary file 2 — Appendix S1.. Supporting Information. [file BJS-107-191-s002.pdf]

PubMed

("Pancreatitis"[Mesh] OR "Pancreas"[Mesh] OR pancrea*[tiab]) AND ("Acute Disease"[Mesh] OR "Recurrence"[MeSH Terms] OR acute*[tiab] OR recurr*[tiab] OR relaps*[tiab]) AND (idiopathic[tiab] OR unexplain*[tiab] OR un-explain*[tiab] OR unknown*[tiab]) AND ("Cholecystectomy"[Mesh] OR "Gallbladder"[Mesh] OR cholecystectom*[tiab] OR remov*[tiab] OR resect*[tiab] OR gallbladder*[tiab]) NOT ("Animals"[Mesh] NOT "Humans"[Mesh]) NOT ("Case Reports" [Publication Type] OR "Letter" [Publication Type] OR "Comment" [Publication Type] OR letter*[ti] OR comment*[ti] OR case report*[tiab] OR rat[tiab] OR rats[tiab] OR mouse[tiab] OR mice[tiab] OR animal*[tiab])

Embase (Ovid)

Database(s): **Embase Classic+Embase** 1947 to 2018 May 01

Search Strategy:

#	Searches
**1**	acute pancreatitis/
**2**	pancreas/ or pancrea*.ti,ab,kw.
**3**	recurrent disease/ or (acute* or recurr* or relaps*).ti,ab,kw.
**4**	2 and 3
**5**	1 or 4
**6**	idiopathic disease/ or (idiopathic or unexplain* or un-explain* or unknown*).ti,ab,kw.
**7**	exp cholecystectomy/ or gallbladder/ or (cholecystectom* or remov* or resect* or gallbladder*).ti,ab,kw.
**8**	5 and 6 and 7
**9**	animal/ not human/
**10**	8 not 9
**11**	editorial/ or letter/ or literature/ or case report/ or (letter* or comment* or rat or rats or mouse or mice or animal*).ti. or case report*.ti,ab,kw.
**12**	10 not 11

Cochrane Central Register of Controlled Trials: Issue 4 of 12, April 2018

ID	Search
#1	MeSH descriptor: [Pancreatitis] explode all trees
#2	MeSH descriptor: [Pancreas] explode all trees
#3	pancrea*:ti,ab,kw (Word variations have been searched)
#4	#1 or #2 or #3
#5	MeSH descriptor: [Acute Disease] explode all trees
#6	MeSH descriptor: [Recurrence] explode all trees
#7	acute* or recurr* or relaps*:ti,ab,kw (Word variations have been searched)
#8	#5 or #6 or #7
#9	idiopathic or unexplain* or un-explain* or unknown*:ti,ab,kw (Word variations have been searched)
#10	MeSH descriptor: [Cholecystectomy] explode all trees
#11	MeSH descriptor: [Gallbladder] explode all trees
#12	cholecystectom* or remov* or resect* or gallbladder*:ti,ab,kw (Word variations have been searched)
#13	#10 or #11 or #12
#14	#4 and #8 and #9 and #13 in Trials
