# Supplementary material for: Recurrence of idiopathic acute pancreatitis after cholecystectomy: systematic review and meta‐analysis
Source: Br J Surg. 2019 Dec 25;107(3):191–9. doi: 10.1002/bjs.11429 (PMC7003758; doi:10.1002/bjs.11429)
Supplement: Supplementary file 3 — Table S1. Assessment of quality according to Newcastle–Ottawa Scale [file BJS-107-191-s003.pdf]

	Selection				Comparability	Outcome		
	Representativeness of the exposed	Selection of the non-exposed cohort	Ascertainment of exposure	Demonstration that outcome of	Comparability of cohorts on the basis of the design or analysis	Assessment of outcome	Was follow-up long enough for	Adequacy of follow up of cohorts
Lee, 1992	**A**	**A**	**A**	B	-	**B**	**A**	D
Pérez-Martin, 1998	**A**	B	**A**	B	-	**B**	B	D
Liu, 2000	**A**	**A**	**A**	B	-	**B**	B	D
Tandon, 2001	C	**A**	**A**	B	-	**B**	B	D
Saraswat, 2004	C	**A**	**A**	B	-	**B**	**A**	C
Garg, 2007	C	**A**	**A**	B	-	**B**	B	**B**
Ortega, 2011	C	**A**	**A**	B	**A**	**B**	B	D
Trna, 2012	C	C	**A**	B	**A+B**	**B**	**A**	D
Räty, 2015	**A**	**A**	**A**	**A**	**B**	**B**	**A**	**A**
Stevens, 2016	**A**	**A**	**A**	B	-	**B**	**A**	**A**

Supplemental file 5: *Assessment of quality according to the Newcastle-Ottawa Scale. "A", "B", "C" and "D" correspond with the scoring for the individual components of the Newcastle-Ottawa Scale. Letters in bold indicate a positive assessment of quality (i.e. resulting in awarding the study with a star) regarding the specific component of the Scale.*
