# Supplementary material for: Recurrence of idiopathic acute pancreatitis after cholecystectomy: systematic review and meta‐analysis
Source: Br J Surg. 2019 Dec 25;107(3):191–9. doi: 10.1002/bjs.11429 (PMC7003758; doi:10.1002/bjs.11429)
Supplement: Supplementary file 4 — Table S2. Components of standard work‐up according to current guidelines and extent to which they were executed in included studies [file BJS-107-191-s004.pdf]

Standard work-up	Lee	Perez-Martin	Liu	Tandon	Saraswat	Garg	Ortega	Trna	Räty	Stevens
**Alcohol**	Men: > 80 g/day; women: > 60 g/day	CAGE (NFS); or > 30 g/day;	Mean > 30 mg/day	Patients with a history of alcohol use but no evidence of CP were included	Alcohol consumption	> 40 g/day for > 5 years	> 80 g/d 2 weeks before admission or chronic alcohol intake	Alcohol abuse	AUDIT > 8; CDT and GT (NFS)	Specific remark that alcohol was not thought to be a causative factor
**Drugs**	Thiazide diuretic, corticosteroid, azathioprine, cyclosporine (A)	Thiazides, valproic acid, sulfamides, tetracycline's, steroids, antineoplastic agents (A)	Drugs known to induce AP (B)	NR (C)	NR (C)	Drugs known to induce AP (B)	Toxic substances or drugs related to the cause of AP in the previous 2 weeks (B)	NR (C)	NR (C)	NR (C)
**Hypercalcemia**	NFS (B)	> 10 mg/dl, repeated (A)	NFS (B)	NR (C)	Abnormal levels, repeated (A)	Repeated, NFS (B)	NFS (B)	Elevated (A)	NFS (B)	NR (C)
**Hypertriglyceridemia**	NFS (B)	> 500 mg/dl, repeated (A)	NFS (B)	Previously documented hyperlipidemia (B)	Abnormal levels, repeated (A)	Repeated, NFS (n = 3) (B)	NFS (B)	Elevated (A)	NFS (B)	NR (C)

Supplemental file 8: components of standard work-up according to current guidelines [11] and the extent in which they were executed in the included studies.

CAGE = Cutting down, Annoyance by criticism, Guilty feeling, Eye openers) questionnaire. NFS = not further specified. CP = chronic pancreatitis. AUDIT = Alcohol Use Disorders Identification Test. CDT = desialotransferrine. GT = glutamyltransferase. AP = acute pancreatitis. NR = not reported. NFS = not further specified. Panels labeled "A" correspond with conduct according to the guidelines, panels labeled "B" correspond with conduct not according to guidelines but pursuance of the particular component of standard work-up or insufficient information to assess adherence to guidelines, and panels labeled "C" correspond with failure to exclude based on the particular component of standard work-up. *Table continues on the next page.*

Standard work-up	Lee	Perez-Martin	Liu	Tandon	Saraswat	Garg	Ortega	Trna	Räty	Stevens
**Detection of biliary etiology**										
**Liver enzymes**	NR (C)	NR (C)	NR (C)	NR (C)	Abnormal AF, repeated (C)	Bilirubin, AF, AST, ALT, NFS (B)	NR (C)	> 3 x ULN of ALT or AST (A)	Elevated ALT, AST, AF, bilirubin (n = 10) (C)	ALT > 150 IU/L (n = 23) (C)
**Gallstones on imaging**	US (A)	US (A)	US (A)	Imaging studies (B)	US (15), CT (9) (B)	US (A)	Conventional imaging explorations (B)	Gallstones in the gallbladder, NFS (B)	US (A)	US (A)
**CBD dilatation on imaging**	US (A)	US (A)	NR (C)	NR (C)	NR (C)	NR (C)	NR (C)	NR (C)	NR (C)	NR (C)
**Sludge on imaging**	NR (C)	US (A)	NR (C)	Imaging studies (B)	NR (C)	US (A)	NR (C)	NR (C)	NR (C)	US (n = 14) (C)
**Repeat ultrasound**	Yes (A)	Yes (A)	Performed in 9/18 patients (B)	Performed in part (B)	Yes (A)	Yes (n = 3) (A)	NR (C)	NR (C)	Yes (A)	NR (C)

Supplemental file 8 (continued): components of standard work-up according to current guidelines [11] and the extent in which they were executed in the included studies.

Panels labeled "A" correspond with conduct according to the guidelines, panels labeled "B" correspond with conduct not according to guidelines but pursuance of the particular component of standard work-up or insufficient information to assess adherence to guidelines, and panels labeled "C" correspond with failure to exclude based on the particular component of standard work-up. NR = not reported. AF = alkaline phosphatase. AST = aspartate transaminase. ALT = alanine transaminase. NFS = not further specified. ULN = upper limit of normal. AP = acute pancreatitis. US = transabdominal ultrasound. CT = computed tomography. Table continues on the next page.

Standard work-up	Lee	Perez-Martin	Liu	Tandon	Saraswat	Garg	Ortega	Trna	Räty	Stevens
**Patients with "original" IAP (n)**	29	18	18	31	24	75	49	23	85	195
**Patients with demonstrable etiology in standard work-up (n)**	0	0	0	0	0	8	0	0	10	34

Supplemental file 8 (continued): components of standard work-up according to current guidelines [11] and the extent in which they were executed in the included studies.

Garg et al. was the only to exclude patients with abdominal trauma or surgery in the 3 months previous to the admission for AP. Ortega et al. were the only to exclude patients based on positive family history for acute pancreatitis. Räty et al. did not exclude the 2 patients with a positive family history for acute pancreatitis but did test for SPINK-1 and PRSS-1 mutations (which were negative). None of the included studies reported on exclusion of post-ERCP pancreatitis, but Liu et al., who excluded acute pancreatitis induced by ERCP, performed for indications unrelated to stone disease. Garg et al. included 3 patients with hypertriglyceridemia, 2 with biliary stones and 1 with chronic pancreatitis on repeat ultrasound, and 2 patients with a positive family history for chronic pancreatitis.
