# Supplementary material for: Recurrence of idiopathic acute pancreatitis after cholecystectomy: systematic review and meta‐analysis
Source: Br J Surg. 2019 Dec 25;107(3):191–9. doi: 10.1002/bjs.11429 (PMC7003758; doi:10.1002/bjs.11429)
Supplement: Supplementary file 5 — Table S3. Additional diagnostic work‐up [file BJS-107-191-s005.pdf]

Study	Lee	Pérez-Martin	Liu	Tandon	Saraswat	Garg	Ortega	Trna	Räty	Stevens
**CT performed (n)**	"most"	NR	6	30	NR	"patients with recurring attacks"	NR	NR	56	NR
**Positive results of CT**	0	NA	0	0	NA	CP: 17	NA	NA	0	NA
**ERCP performed (n)**	"most"	NR	13	1	24	52	NR	NR	NR	NR
**Positive results of ERCP**	0	NA	0	Malignancy: 1	0	CP: 18<br>Divisum: 4<br>Other: 2	NA	NA	NA	NA
**Bile examination performed (n)**	29	17	NR	13	24	52	1	NR	NR	NR
**Positive for biliary cause**	21	13	NA	3	18	20	1	NA	NA	NA
**EUS performed (n)**	NR	NR	18	31	NR	10	49	NR	0	0
**Positive results of EUS**	NA	NA	Biliary: 14	Biliary: 2<br>CP: 14<br>Divisum: 2<br>Pancreatic mass: 1	NA	CP: 7	Biliary: 15<br>CP: 8<br>Other: 3	NA	0	0
**MRCP performed (n)**	NR	NR	NR	1	NR	NR	49	NR	28	48
**Positive results of MRCP**	NA	NA	NA	Divisum: 1	NA	NA	Biliary: 4<br>Divisum: 4<br>Other: 2	NA	0	Sludge: 5<br>Other: 1
**Patients with demonstrable etiology after additional work-up**	21	13	14	21	18	49	29	0	0	0
**Patients with "true" IAP**	8	5	4	10	6	18	20	23	75	195

Supplemental file 9: additional diagnostic work-up. *CT = computed tomography. ERCP = endoscopic retrograde cholangiopancreaticography. NR = not reported; NA = not applicable; CP = chronic pancreatitis. EUS = endoscopic ultrasound. MRCP = magnetic resonance cholangiopancreaticography. Other findings of ERCP include ascariasis (n=1) and choledochal cyst (n=1) [4]. Other findings on EUS include divisum (n=1), intraductal papillary mucinous neoplasm (n=1), and choledochocele (n=1) [20]. Other findings of MRCP include CP (n=1), IPMN (n=1) [20] and a gallbladder polyp (n=1) [8]. This includes at least 34 patients included by Stevens et al., in whom sludge was found on transabdominal ultrasound, elevated ALT levels were measured, sludge was found on MRCP or a combination of these indicators. Due to insufficient information on what amount of patients had a combination of these abnormalities, the patients with a high probability of a biliary etiology could not be deducted.*
